# The Adapted Firesetting Assessment Scale: reliability and validity

**DOI:** 10.1111/jir.12950

**Published:** 2022-05-27

**Authors:** J. Collins, P. E. Langdon, M. Barnoux

**Affiliations:** ^1^ Tizard Centre University of Kent Canterbury UK; ^2^ Centre for Educational Development, Appraisal and Research (CEDAR) The University of Warwick Coventry UK; ^3^ Coventry and Warwickshire Partnership NHS Trust Coventry UK

**Keywords:** AFAS, Arson, autism, developmental disabilities, firesetting, intellectual disabilities

## Abstract

**Background:**

The Adapted Firesetting Assessment Scale was developed for use with adults with developmental disabilities targeting fire‐related factors thought to be associated with deliberate firesetting behaviour (i.e. attitudes towards fire, fire interest, fire normalisation, identification with fire and fire safety awareness). However, the psychometric properties of the scale are yet to be evaluated.

**Method:**

The reliability, validity, comprehensibility, relevance and comprehensiveness of the Adapted Firesetting Assessment Scale were evaluated. Fifty‐nine adults with developmental disabilities, some of whom had a history of firesetting, completed the Adapted Firesetting Assessment Scale on two occasions. Feedback about the questionnaire was sought from both participants and professionals.

**Results:**

The AFAS has acceptable internal consistency and excellent test–retest reliability. The attitudes towards fire, fire normalisation, poor fire safety subscales and total scores discriminated firesetters from non‐firesetters. Content analysis of feedback indicated items of the AFAS were understood, relevant, accessible and comprehensible.

**Conclusion:**

A larger study is needed to examine the factor structure of the AFAS.

## Introduction

Within England and Wales, deliberate firesetting is estimated to cost £1.45 billion per year (Arson Prevention Forum 2017), with 63 712 incidents of deliberate firesetting reported for the period 2020 to 2021, which resulted in 59 deaths and 880 non‐fatal causalities (Home Office 2021). Firesetting among adults with developmental disabilities and more specifically with intellectual disabilities (IDs), autism or both is consistently reported within the literature (e.g. Simpson & Hogg 2001; Lees‐Warley & Rose 2015; Collins *et al*. 2021b).

Consequently, several assessments have been developed, adapted or validated for use with adults with developmental disabilities who set fires to target specific treatment needs identified as relevant. Current assessments relate to self‐esteem/self‐efficacy (e.g. Adapted Rodenberg Self‐Esteem Scale; Dagnan & Sandhu 1999), emotional regulation skills (e.g. Modified Overt Aggression Scale; Kay *et al*. 1988), interpersonal relationships and social skills (e.g. Rathus Assertiveness Scale; Rathus 1973) and psychopathology (e.g. Psychiatric Assessment Schedule for Adults with Developmental Disabilities‐Shortened version; Moss *et al*. 1993).

Unlike other factors associated with firesetting behaviour, fire‐related factors (including serious fire interest, identification with fire, normalisation of fire and poor fire safety awareness) are associated with an increased risk of firesetting and have been shown to discriminate those without developmental disabilities who do and who do not set fires (MacKay *et al*. 2006; Gannon *et al*. 2013; Ó Ciardha *et al*. 2014; Tyler *et al*. 2015). Fire‐related factors have also been integrated into our theoretical understanding of firesetting behaviour for people without developmental disabilities and conceptualised as reinforcement contingencies (Fineman 1995), psychological vulnerabilities (e.g. Gannon *et al*. 2012) and key risk factors (e.g. Tyler *et al*. 2014). People without developmental disabilities who set fires have self‐reported higher levels of serious fire interest, normalisation of fire, identification with fire and lower levels of fire safety awareness (Clare *et al*. 1992; Taylor *et al*. 2002; Haines *et al*. 2006; Dickens *et al*. 2009; Gannon *et al*. 2013; Barnoux *et al*. 2015; Gannon *et al*. 2015). Furthermore, several other studies have suggested that experiencing heightened excitement around fire represents a risk factor for repeat firesetting behaviour (MacKay *et al*. 2006; Dickens *et al*. 2009).

In addition, a fascination and attraction to fire, along with affective arousal before a fire is set, is part of the diagnostic criteria for pyromania in the Diagnostic and Statistical Manual of mental Disorders (American Psychiatric Association 2013). The prevalence rates of pyromania vary between 0% and 10% among those with a history of firesetting known to the criminal justice system at pre‐trial examination, hospital or prison. A variation in prevalence is perhaps due to differences in research designs including data collection strategies and differences between participant samples (Geller & Bertsch 1985; O'Sullivan & Kelleher 1987; Ritchie & Huff 1999; Lindberg *et al*. 2005). The low prevalence of pyromania among those who set fires might be attributable to the strict diagnostic criteria, whereby alongside a series of specific indicators of fire interest (e.g. pleasure and relief upon setting a fire), pyromania is diagnosed only in the absence of all other motivators such as antisocial personality disorder, alcohol, delusions and other common motivators of firesetting (e.g. revenge or anger).

Nevertheless, existing research has suggested that fire interest or a fascination with fire represents one factor likely to increase firesetting risk (Jackson *et al*. 1987; Fineman 1995; MacKay *et al*. 2006; Dickens *et al*. 2009). However, the strength of the association between the fire‐related factors and deliberate firesetting by people with developmental disabilities has not been adequately investigated. Although, there is tentative evidence to suggest some adults with developmental disabilities who set fires do have a special interest in fire (e.g. Radley & Shaherbano 2011; Holst *et al*. 2019; Collins *et al*. 2021b).

The Fire Interest Rating Scale (Murphy & Clare 1996) and the Firesetting Assessment Schedule (Murphy & Clare 1996) are the only measures known to have been specifically developed for adults with IDs that focus on fire‐related factors associated with offending behaviour. There are issues with the lack of information about their reliability, validity and clinical utility due to a lack of psychometric evaluation. Murphy and Clare (1996) suggested the Fire Interest Rating Scale lacked discriminative validity and respondents were required to have good verbal skills and an ability to label emotions, therefore excluding adults with more severe impairments. Also, having been developed in 1996, these measures predate more recent advancements in the field and have not been developed for use with autistic adults.

Several other measures not developed for adults with developmental disabilities could be used to assess fire‐related factors including the Identification with Fire Questionnaire (Gannon *et al*. 2011). However, items might be more challenging for this population to answer due to known deficits in abstract reasoning (Solomon *et al*. 2011). The Four‐Factor Fire scale (Ó Ciardha *et al*. 2015), which combines items from the Fire Attitudes Scale (Muckley 1997), the Identification with Fire Questionnaire (Gannon *et al*. 2011) and the Fire Interest Rating Scale (Murphy & Clare 1996), has been used in practice when assessing adults who set fires for treatment suitability and therapeutic evaluation (Gannon *et al*. 2013; Gannon *et al*. 2015). However, research does not support the validity of these measures when used with adults with developmental disabilities, therefore limiting our knowledge and understanding of firesetting behaviour among this population.

Therefore, an adapted scale grounded in the most recent research on the fire‐related factors identified in the literature may support clinicians in the assessment and treatment of adults with developmental disabilities who set fires. Informed by the Four‐Factor Fire scale (Ó Ciardha *et al*. 2014), expert and service‐user opinion, the Adapted Firesetting Assessment Scale (AFAS; Collins *et al*. 2021a) was developed for use with adults with developmental disabilities targeting factors related to fire that are thought to be associated with deliberate firesetting behaviour (i.e. attitudes towards fire, fire interest, fire normalisation, identification with fire and fire safety awareness). However, the psychometric properties of the scale have not been evaluated.

The aims of the current study were to investigate the reliability, validity, comprehensibility, relevance and comprehensiveness of an adapted self‐report measure for adults with developmental disabilities that aimed to assess fire interest, fire normalisation, fire safety awareness and identification with fire.

## Method

### Participants

A total of 59 participants (26 firesetters and 33 non‐firesetters) using a purposive sampling method were recruited from psychiatric hospitals in England specialising in the care and treatment of adults with developmental disabilities, which included low secure units (*n* = 29), medium secure units (*n* = 21) and locked rehabilitation units (*n* = 5) spread across seven different sites. Four participants were recruited from the community (1 firesetter and 3 adults without an offending history), also in England. Participants were included if they were 18 years or above, diagnosed with either ID and/or autism by a clinical psychologist, psychiatrist or other appropriately qualified professional. Participants were allocated to the firesetter group if they had a history of deliberate firesetting defined as an index offence for Arson, or previous conviction for Arson, or an incident of deliberate firesetting recorded in their case records. Participants were excluded if they had an inability to give or withhold consent to take part as defined within the Mental Capacity Act (2005). Of the 59 participants who completed the AFAS at Time 1, 56 completed the measure a second time leading to an attrition rate of 5%.

The full‐scale IQ was obtained for 38 participants (*M* = 64.95, *SD* = 14.53). All participants had a diagnosed developmental disability, including ID (*n* = 46), autism (*n* = 25) and/or other developmental disability, including Klinefelter's syndrome, attention deficit hyperactivity disorder and hyperkinetic disorder (*n* = 10). Adults with a range of developmental disabilities were included in the research due to the high rates of comorbidity, with as many as 40% of children and young people with IDs also diagnosed as autistic (Kinnear *et al*. 2019). Furthermore, the commonalities between developmental disabilities (e.g. social communication difficulties) have previously led to difficulties differentiating autism and IDs (Thurm *et al*. 2019). We therefore adopted an inclusive approach to sampling by including those with IDs, autism or both.

### Measures

The AFAS (Collins *et al*. 2021a) is a self‐report measure of the fire‐related factors (i.e. fire interest, fire attitudes, normalisation of fire, poor fire safety and identification with fire). The AFAS is composed of 41 items, 27 of which are rated on a 3‐point Likert scale (1 = *Agree*, 2 = *Not Sure*, 3 = *Disagree*) and 14 items are rated on a 4‐point Likert scale (1 = *Very upset/scared*, 2 = *Little upset/scared*, 3 = *Ok*, 4 = *Excited/Fun*). Items 1, 3–11, 14–15, 17–19, 21–23, 25–26 and 41 are reverse scored. A total score is generated by summing the individual item scores and dividing the total by the number of items answered. A higher value on the AFAS is indicative of more problematic attitudes and beliefs towards fire, serious fire interest and identification with fire, and poor fire safety knowledge. The items forming the AFAS are found in Table 1.

**Table 1:** Items of the AFAS

Item no.	AFAS item (subscale(s) in which the item appears)
1	Fire is very important to me. (IS; 4‐Factor IS)
2	I would be happy if I never saw a fire again. (IS)
3	Fire is a big part of my life. (IS; 4‐Factor IS)
4	I need fire in my life. (IS; 4‐Factor IS)
5	I would describe myself as someone who starts fires. (IS; 4‐Factor IS)
6	I am nobody without fie (e.g. nobody notices me). (IS; 4‐Factor IS)
7	I must have fire in my life. (IS; 4‐Factor IS)
8	Most people carry a lighter with them. (FA)
9	People often set fires when they are angry. (FA)
10	I would like to work as a firefighter. (FA)
11	I like watching fires get bigger. (FA; 4‐Factor IS)
12	I have put a fire out. (FA)
13	They should teach you how to stop fires at school. (FA; 4‐Factor PFS)
14	Most people's friends have started a fire or two. (FA; 4‐factor NF)
15	The police talk to lots of people about setting fires. (FA; 4‐factor NF)
16	I know a lot about how to stop a fire. (FA; 4‐Factor PFS)
17	Setting a small fire can make you feel better. (FA; 4‐Factor IS)
18	I can stop a fire from getting too big. (FA; 4‐Factor PFS)
19	I get bored easily. (FA; 4‐factor NF)
20	People who set fires should be sent to prison. (FA)
21	I often copy what my friends do without thinking. (FA; 4‐factor NF)
22	If you have problems, a small fire can help you sort them out. (FA; 4‐Factor IS & PFS)
23	Most people have had an accident at home/in hospital that involved fire. (FA; 4‐factor NF)
24	Parents/carers should spend money on buying a fire extinguisher. (FA; 4‐Factor PFS)
25	Most people have set a small fire for fun. (FA; 4‐factor NF)
26	I usually copy what my friend do. (FA; 4‐factor NF)
27	Playing with a lighter can be dangerous. (FA; 4‐Factor PFS)
28	Having a lighter in my pocket. (FI)
29	Watching fire burn in a fireplace. (FI)
30	Watching a bonfire on fireworks night. (FI)
31	Seeing a firefighter put their uniform on (e.g. helmet) (FI)
32	Watching a fire engine come down the road. (FI)
33	Using a lighter to start a cigarette. (FI)
34	Watching a house burn down. (FI; 4‐Factor FI)
35	Being questioned by the police about a fire that has happened in the neighbourhood. (FI; 4‐Factor FI)
36	Watching people run from a fire. (FI; 4‐Factor FI)
37	Watching a person with their clothes on fire. (FI; 4‐Factor FI)
38	Using a lighter to set fire to a building. (FI; 4‐Factor FI)
39	Seeing a building on fire in the news. (FI; 4‐Factor FI)
40	Seeing a firefighter use water to put a fire out. (FI; 4‐Factor FI)
41	Giving a lighter back to someone (FI)

4‐Factor, Four‐Factor Fire Scale; AFAS, Adapted Firesetting Assessment Scale; FA, attitudes towards fire subscale; FI, fire interest subscale; IS, identification with fire subscale; NF, fires as normal subscale; PFS, poor fire safety subscale.

### Design and procedure

Using a cross‐sectional and between‐subjects design, adults with developmental disabilities who did or did not have a history of firesetting were invited to take part in this study and complete the AFAS on two occasions, approximately 2 weeks for most participants (min = 6, max = 128 days, *M* = 18.48 days, SD = 16.24). Two weeks is considered the most suitable timeframe as a very short time interval between testing, such as 1 week, might lower the risk that an attribute has changed, but it increases the risk that respondents will remember the questions and their answers (Polit 2014). This study was conducted during the global pandemic between 2020 and 2021 when many services in the United Kingdom were in lockdown impacting on the consistency across participants for the time between the first and second rounds of data collection. Participants self‐reported their age, ethnicity, full‐scale IQ, index offence, previous offences and mental health diagnosis (where applicable). For participants known to services with a clinical care team (*n* = 56), their informed consent was obtained to check these details with their responsible clinician. Detail of information obtained from participants in the community and inpatient services was comparable, although two of the four community participants could not provide their FSIQ score.

The study received a favourable ethical opinion from an NHS Research Ethics Committee and Health Research Authority approval (IRAS ID: 255255), people detained within hospitals were informed about the study and invited to speak to a member of the team to consider whether they would like to participate. They were provided with an information sheet explaining the study and those who wished to take part were asked to sign a consent form. The study was also advertised on social media platforms (i.e. Twitter and Facebook). People in the community were encouraged to contact the researcher via email if they wished to express their interest in participating in the study. If an individual was eligible to participate, an information sheet and consent form was sent via email. Completed consent forms were returned to the researcher before a virtual meeting was arranged. Upon receiving informed consent, the researcher met with individual participants using Zoom video conferencing software, and the AFAS was shared with the participant who indicated their response verbally to the researcher.

Participants and practitioners were also asked to complete a short feedback form comprised of four questions to provide some evaluation of the comprehensibility, relevance, comprehensiveness and usefulness of the AFAS (Mokkink *et al*. 2018; Prinsen *et al*. 2018; Terwee *et al*. 2018). Practitioners were qualified professionals working with adults with developmental disabilities who set fire (e.g. assistant psychologists, psychiatrist and psychologists). Questions to participants included, ‘Were the questions easy to understand’, ‘Were the questions relevant to autistic adults and adults with IDs who set fires’, ‘Did the pictures support your understanding of the written text’ and ‘Did the response options make sense?’ Questions to practitioners included, ‘Would you use the questionnaire as part of your assessment of an adult with IDs and/or autism who presents with firesetting behaviour’, ‘Do you think the questions were relevant to adults with IDs and/or autism who have set a fire’, ‘Was the questionnaire useful in assessing adults with intellectual and other developmental disabilities who may/may not have a history of firesetting behaviour’ and ‘Were the response options adequate?’ Participants and practitioners answered the four questions ticking either ‘yes’ or ‘no’, and space was provided for further comments.

### Data analysis

The current analysis was conducted using both the original subscales (i.e. fire interest, identification with fire and attitudes towards fire), as well as the subscales of the Four‐Factor Fire Scale (i.e. fire interest, fire as normal, identification with fire and poor fire safety), and the total scores for each. The analysis was conducted on both the original subscales and for items included in the four subscales outlined by Ó Ciardha *et al*. (2014) to allow for an investigation into the validity of the scale bearing in mind that this instrument had not been previously used with this population. It was anticipated that the test–retest reliability, internal consistency and validity of the instrument may be superior when scored using the four factors outlined by Ó Ciardha *et al*. (2014) as opposed to the original scales.

### Missing data

Of the 59 participants, 58 completed all 41 items. One participant completed 40 items at Time 1, which was <1% of all items completed at Time 1 and Time 2. Summed scores on the AFAS were adjusted by dividing the summed score by the number of completed items to generate a total score accounting for missing responses.

### Internal consistency

Internal consistency of the AFAS was determined by calculating Cronbach's *α* coefficient. Alternative measures of internal consistency were considered (Viladrich *et al*. 2017). However, evidence on their use is inconsistent and not well developed. Authors do however acknowledge the limitations associated with relying on Cronbach's *α* to determine the internal consistency of a scale as it is assumed the scale or subscale is unidimensional (Cho & Kim 2015). For the current study, it cannot be suggested that the items measure the same latent construct (Leppink & Perez‐Fuster 2017). Nevertheless, due to the small sample size, *α* was calculated on the scale as a precursor for future factor analysis.

Internal consistency was calculated on the first (*n* = 59) and the second completion for each participant (*n* = 56). The Consensus‐Based Standards for the Selection of Health Measurement Instruments manual suggests good internal consistency is indicated by an alpha value ≥ 0.70 (Mokkink *et al*. 2018; Prinsen *et al*. 2018; Terwee *et al*. 2018).

### Test–retest reliability

Test–retest reliability is a measure of stability and is used to determine the extent to which items on a measure are consistent and replicable. It is the only way to demonstrate the similarities between responses to items provided by participants on at least two different occasions (Leppink & Perez‐Fuster 2017). Interclass correlations and their 95% confidence intervals were calculated based on absolute‐agreement, two‐way random model, to investigate test–rest reliability for both Time 1 and Time 2 total scores based on the sum of all included items divided by the total number of items answered (i.e. the original subscales) and again based on the Four‐Factor Fire Scale as interclass correlations are sensitive to the detection of systematic error and reflect both degree of correlation and agreement between measurements (Koo & Li 2016).

### Discriminative validity

Discriminative validity was investigated by comparing AFAS scores between firesetters and non‐firesetters using *t*‐tests or Mann–Whitney *U*, depending on whether the data were normally distributed. The fire interest subscale and the attitudes towards fire subscale derived from the original three measures and fire normalisation subscale derived from the Four‐Factor Fire Scale were normally distributed, but this was not the case for the remaining subscale scores and the total scores as indicated by histograms that depicted positively skewed distributions.

### Participant and practitioner feedback

The percentage of participants and practitioners who responded either yes or no to each question was summarised. A fifth question asked for any additional comments. A total of 59 participants and 13 practitioners, representing all seven research sites participating in the study, completed the feedback form. Content analysis was used to analyse the qualitative feedback, whereby comments made by participants and practitioners were coded and categorised systematically into either *positive comment about the AFAS*, *comment that suggested the AFAS required improvement*, or *other comments* based on the descriptive words and context. A second independent researcher openly coded all comments, leading to 100% agreement. Of the 43 comments made by participants and practitioners, four were separated into two components by Rater 1 and not by Rater 2. Agreement that both components should be categorised individually was reached through discussion between raters.

## Results

Participant demographics for each of the two groups (i.e. firesetters and non‐firesetters) are reported in Table 2. Firesetters and non‐firesetters did not differ significantly on FSIQ scores, *z* = −0.37, *P* = 0.71, *r* = 0.06. However, firesetters had a significantly lower age; *z* = 1.96, *P* = 0.05, *r* = 0.3, and higher number of previous convictions compared with non‐firesetters; *z* = −2.12, *P* = 0.03, *r* = 0.3. There was no significant association between firesetting status and gender, *χ* ^2^ (2, *n* = 59) = 1.93, *P* = 0.38, ɸ = 0.16, ethnicity, *χ* ^2^ (2, *n* = 59) = 3.61, *P* = 0.16, ɸ = 0.21, and type of service, *χ* ^2^ (5, *n* = 59) = 9.53, *P* = 0.09, ɸ = 0.37.

**Table 2:** Participant demographics

Demographics	Firesetters (*n* = 26)	Non‐firesetters (*n* = 33)
Age in years	*M* = 33.04* (SD = 11.38)	*M* = 37.67 (SD = 11.06)
Gender		
Males	23 (88.5%)	29 (87.9%)
Females	2 (7.7%)	4 (12.1%)
Transgender	1 (3.8%)	0
Index offence		
Violence	8 (30.8%)	13 (39.4%)
Property	1 (3.8%)	2 (6.1%)
Sexual	6 (23.1%)	11 (33.3%)
Arson	7 (26.9%)	0
None	4 (15.4%)	7 (21.2%)
Number of previous offences	*M* = 6.46* (SD = 10.64)	*M* = 2.36 (SD = 2.76)
FSIQ	*M* = 63.82, SD = 7.63 (*n* = 17)	*M* = 65.86, SD = 18.49 (*n* = 21)
Ethnicity		
White UK/Irish	26 (100%)	30 (90.9%)
Pakistani	0	1 (3%)
Black Caribbean	0	2 (6.1%)
Type of service		
Low secure unit	10 (38.5%)	19 (57.6%)
Medium secure unit	11 (42.3%)	10 (30.3%)
Locked rehabilitation	4 (15.4%)	1 (3%)
Community	1 (3.8%)	3 (9.1%)
Diagnosis		
Intellectual disability	22 (84.6%)	26 (78.8%)
Autism	10 (38.5%)	15 (45.5%)
Other developmental	7 (26.9%)	4 (12.1%)
Affective	1 (3.8%)	3 (9.1%)
Personality disorder	10 (38.5%)	5 (15.2%)
Conduct disorder	1 (3.8%)	1 (3%)
Psychosis	5 (19.2%)	6 (18.2%)
Substance misuse	1 (3.8%)	1 (3%)
Speech impediment	1 (3.8%)	0
OCD	1 (3.8%)	1 (3%)
Schizoaffective disorder	2 (7.7%)	1 (3%)
Chronic fatigue	1 (3.8%)	0
Paedophilia	1 (3.8%)	0
Fetishistic disorder	1 (3.8%)	0

* *P* < 0.05.

### Internal consistency

Findings suggested that the AFAS overall has acceptable internal consistency (DeVellis & Thorpe 2021). The value for Cronbach's *α* for individual subscales varied between 0.60 and 0.87, except for the poor fire safety subscale, which had very poor internal consistency (refer to Table 3).

**Table 3:** Internal consistency of the Adapted Firesetting Assessment Scale

Original scales	Cronbach *α*	
	Time 1 (*n* = 59)	Time 2 (*n* = 56)
Identification subscale	0.82	0.83
Fire interest subscale	0.68	0.60
Fire attitudes subscale	0.71	0.71
Full scale	0.81	0.81
Four‐Factor Fire Scale		
Identification subscale	0.85	0.87
Poor fire safety subscale	0.16	−0.33
Fire as normal subscale	0.72	0.70
Serious fire interest subscale	0.74	0.79
Full scale	0.82	0.84

### Test–retest reliability

Interclass correlation coefficient estimates indicated excellent reliability between the total score across time for the original, ICC(56) = 0.91, 95% confidence interval [0.86, 0.95], and Four‐Factor Fire Scales, ICC(56) = 0.93, 95% confidence interval [0.87, 0.96].

### Discriminative validity

#### Original scales

Those with a history of firesetting scored significantly higher on the attitudes towards fire subscale, *t*(57) = 2.86, *P* < 0.01 and higher on the AFAS total score, *z* = −0.21, *P* = 0.03 suggesting they had more problematic attitudes towards fire. There was no significant difference between the groups on the fire interest subscale, *t*(57) = 1.79, *P* = 0.08 or identification with fire subscale, *z* = −0.01, *P* = 0.99, Table 4.

**Table 4:** Descriptive statistics of adjusted total scores for Time 1

	Firesetters (*n* = 26)			Non‐firesetters (*n* = 33)		
	*M* = (SD)	Mdn	Min; Max	*M* = (SD)	Mdn	Min; Max
Original scales						
Identification subscale	1.46 (0.51)	1.29	1.00; 2.71	1.46 (0.54)	1.29	1.00; 2.86
Fire interest subscale	2.38 (0.42)	2.39	1.57; 3.43	2.21 (0.29)	2.14	1.50; 2.71
Fire attitudes subscale*	1.86 (0.29)	1.83	1.30; 2.60	1.65 (0.29)	1.60	1.20; 2.55
Total score Time 1*	1.97 (0.28)	1.90	1.56; 2.90	1.81 (0.24)	1.80	1.34; 2.46
Four‐Factor Fire Scale						
Identification subscale	1.44 (0.54)	1.22	1.00; 2.78	1.41 (0.48)	1.22	1.00; 2.89
Poor fire safety subscale*	1.62 (0.33)	1.50	1.33; 2.33	1.39 (0.28)	1.33	1.00; 2.33
Fire as normal subscale*	2.18 (0.45)	2.14	1.00; 2.71	1.85 (0.51)	1.86	1.00; 3.00
Serious fire interest subscale	1.90 (0.59)	1.86	1.00; 3.86	1.66 (0.42)	1.57	1.00; 2.57
Total score Time 1*	1.77 (0.35)	1.68	1.21; 2.93	1.59 (0.28)	1.57	1.11; 2.29

* *P* < 0.05 (significant difference between firesetters and non‐firesetters).

##### Four‐Factor Fire scale/subscale

Those with a history firesetting scored significantly higher on the fire normalisation subscale, *t*(57) = −2.58, *P* = 0.01, poor fire safety subscale, *z* = −2.52, *P* = 0.01, *r* = 0.33 and total score, *z* = −2.14, *P* = 0.03, *r* = 0.28 suggesting firesetters were more likely to perceive firesetting as normal and were more likely than non‐firesetters to have a poor knowledge of fire safety. There was no significant difference between the groups on the identification with fire subscale, *z* = −0.08, *P* = 0.94, *r* = 0.00 or fire interest subscale, *z* = −1.69, *P* = 0.09, *r* = 0.22, Table 4.

### Participant feedback

All participants provided feedback on the AFAS following completion (refer to Table 5). On occasions, adults with developmental disabilities did require support to complete items. For example, one respondent stated, ‘Easy to understand but might need support’, and another reported ‘Some questions were difficult because the wording was hard to understand’. This reflects the individual needs of adults with developmental disabilities and as one participant reports, ‘One or two questions might need adjusting for people who are more or less able to understand more complex sentences’. Similarly, another respondent reported ‘Some questions were easy to understand, some were not, some questions could give more information. For example, why you have a lighter or how big the fire is or the occasion’. This may indicate more contextual information is required for these items.

**Table 5:** Participant and practitioner feedback

Questions	Response (%)	
	Yes	No
Participant (*n* = 59)		
Were the questions easy to understand?	93.2	6.8
Were the questions relevant to autistic adults and adults with intellectual disabilities who set fires?	89.8	10.2
Did the pictures support your understanding of the written text?	94.9	5.1
Did the response options make sense	96.6	3.4
Practitioner (*n* = 13)		
Would you use the questionnaire as part of your assessment of an adult with intellectual disabilities and/or autism who presents with firesetting behaviour?	85.7	14.3
Do you think the questions were relevant to adults with intellectual disabilities and/or autism who have set a fire?	92.9	7.1
Was the questionnaire useful in assessing adults with intellectual and other developmental disabilities who may/may not have a history of firesetting behaviour?	78.6	21.4
Were the response options adequate?	85.7	14.3

Other respondents commented positively about the AFAS. For example, one autistic person without intellectual disabilities reported the AFAS was ‘Easy to understand, colour good, order of icons, wording was good. About right with images and words. As an autistic person without intellectual disabilities, I did not find the questionnaire too simple’. Other positive feedback referred to the response options as represented by one participant comment, ‘Emojis made it easier to understand and more fun to do’. Further comments were indicative of formatting changes (e.g. larger images). Feedback categorised as *other comments* included comments that were not directly related to the validity of the AFAS, for example one participant reported ‘I like fire’, and another ‘I am not an arsonist’.

### Practitioner feedback

A total of 13 practitioners representing all seven research sites participating in the study completed the feedback form (refer to Table 5) and 10 provided further comments in the form of qualitative feedback. Of 17 comments made by the 10 professionals, five were categorised as *positive comments about the AFAS* and 12 comments were categorised as *AFAS requires improvement*. Feedback suggested autistic service users found items that were more abstract or those that require some perspective taking more challenging, including ‘I am nobody without fire e.g., nobody notices me’ and ‘Using a lighter to set fire to a building’. Two professionals reported that the ‘questions were leading’. Another four professionals felt amendments to the response options could enhance the scale, including one practitioner who felt having a broader range of emotional responses (e.g. angry) would be beneficial. Although a second reported three rather than four responses would have been better throughout. It could be that the contradictory feedback regarding the response options available is reflective of the service user needs and might indicate the AFAS is more suitable for some adults with developmental disabilities and not others (i.e. those with mild, moderate or severe intellectual disabilities). Professionals also reported that respondents referred to socially acceptable firesetting behaviour, such as BBQ fires. This may suggest some contextual information and guidance to support the administration of the AFAS would be beneficial.

Feedback highlighted the AFAS as a useful tool to use alongside other measures when assessing an adult with a history of deliberate firesetting. One professional commented ‘We already have some familiarity with the original version of the assessments having used them for several years. We judged the materials to be suitable for adults with ID. However, the enhancements improve comprehension’ and another reported that it was ‘Really easy to use and response items meant the service user could fill in more independently’.

## Discussion

### Reliability and validity of the AFAS

The findings indicated that the internal consistency of the AFAS was acceptable, except for the fire safety subscale that was very poor. The AFAS had excellent test–retest reliability and the scale was able to discriminate between firesetters and non‐firesetters, recognising that firesetters had more problematic attitudes towards fire. Firesetters scored higher relative to non‐firesetters on the fire normalisation subscale, had poor fire safety subscale and had a higher total score.

Contrary to expectation, only 34.6% of firesetters with developmental disabilities agreed with the statement, ‘I can stop a fire from getting too big’ (Item 18) suggesting adults with developmental disabilities have knowledge that a fire can be difficult to control. Interesting, a higher percentage of participants without a history of firesetting rated the statement highly in agreement (60.6%) suggesting a lack of knowledge regarding how easily and quickly a fire can spread. The direction of scores for this item is reflected in the negative value for the internal consistency for the poor fire safety awareness subscale. Item 18 did not discriminate firesetters and non‐firesetters for the current sample. Further validation work is required to better understand these findings. However, it may be that firesetters were more experienced having had observed fire spread or become uncontrollable.

Interestingly, the fire interest and identification with fire subscales of the AFAS did not discriminate firesetters from non‐firesetters and may indicate that these fire‐related factors are not as prominent in this population. These findings are supported by recent research in which fire‐related factors were less prominent in the offence chains of adults with developmental disabilities (Collins *et al*. 2021c). Findings suggested adults with developmental disabilities and a history of firesetting scored significantly higher than adults with a developmental disability and no history of firesetting on subscales related to attitudes towards fire, fire normalisation and poor fire safety. These findings may suggest these areas are key to consider during the assessment and treatment of this population and therefore require further exploration. The current study also provided some support for the use of items included in the Four‐Factor Fire Scale over the use of items included in the original measures. With the exception of the poor fire safety subscale, the Four‐Factor Fire Scale had better internal consistency and overall better test–retest reliability when compared with the original measures. This is perhaps unsurprising given the Four‐Factor Fire Scale was developed and evaluated using factor analytic methods, whereby the four factors and their associated items were identified.

### Use of the AFAS, practitioner and participant feedback

Participant and professional feedback was positive, indicating the AFAS was perceived by stakeholders as useful, relevant and comprehensive. However, the AFAS may not be suitable for all adults with developmental disabilities, and some people may need additional contextual information and support before providing an informed response to all items. The individual needs of service users should be considered when administering an assessment.

### Limitations

This study was conducted during the global pandemic between 2020 and 2021 when many services in the United Kingdom were in lockdown. Findings are therefore limited by a small sample size of 59 participants recruited predominantly from a small number of services supporting adults with developmental disabilities in the United Kingdom. It must be acknowledged that a larger sample, inclusive of people of different genders across a range of settings, would have been more representative of all adults with developmental disabilities who set fires. A larger sample would have allowed for firesetters and non‐firesetters to be matched on key characteristics and more definitive conclusions could be established about the AFAS and its factor structure, reliability and validity. In addition, the time between the first and second rounds of data collection for the purpose of test–retest reliability could have been more consistent but was nevertheless calculated as excellent. Furthermore, the administration of the AFAS by an independent researcher may have led to more accurate outcomes as participants in the current study may have been concerned about a member of staff from their service administering the questionnaire.

### Implications and recommendation

The finding of the current study offers some evidence that fire‐related factors are important to consider during the assessment, formulation and treatment with adults with developmental disabilities as subscales thought to be measuring these factors discriminated firesetters from non‐firesetters, including the focus on attitudes towards fire, cognitions pertaining to firesetting being normal and fire safety awareness. However, other factors might be less relevant, such as serious fire interest, but nevertheless important for some individuals. The findings of the current research highlight the need for a person‐centred approach to the assessment and formulation of treatment needs and risks for adults with developmental disabilities who have set a fire. Nevertheless, the findings of the current study suggested that in addition to autistic adults, those with intellectual disabilities may also benefit from further explanations of more abstract concepts. Research could look to explore other methods of assessing these factors that rely less on self‐report (e.g. virtual reality technology, videos, or physiological measures of heart rate and blood pressure), which have been utilised with this population in other areas of assessment rehabilitation (Standen & Brown 2005). Although the AFAS has been adapted for use with adults with developmental disabilities, some additional guidance may prove beneficial to those completing the AFAS and for professionals offering support. It would be useful to collect additional data from professionals delivering the AFAS to service users and service users themselves, exploring the individual items of the AFAS and the time it takes to administer. Additional information regarding questionnaire items and administration would help to ensure it is as useful as possible for professionals. Future studies should seek to evaluate the psychometric properties of the AFAS and explore the uni/multidimensionality of scale items. Nevertheless, findings highlighted the importance of including the target population within the research, seeking the views of the target population, and including both professionals and adults with developmental disabilities within research.

## Conclusions

Further psychometric evaluation of the AFAS is required. Nevertheless, the results of this study indicated that the AFAS appears to have internal consistency, test–retest reliability and discriminative validity. Feedback from professionals and participants suggested that items of the AFAS were comprehensible, relevant, comprehensive and a valuable resource they could utilise in practice to inform their clinical formulations of adults with developmental disabilities who set fires.

## Source of funding

This research did not receive any specific grant from funding agencies in the public, commercial or not‐for‐profit sectors.

## Conflict of Interest

No conflicts of interest have been declared.

## Ethics approval statement

The study was approved by the Health Research Authority and Social Care Research Ethics Committee (IRAS 255255, REC ref: 19/IEC08/0019).

## Data Availability

Data are available by request to the first author.
